# Alternative methods in toxicology: pre-validated and validated methods

**DOI:** 10.2478/v10102-011-0018-6

**Published:** 2011-09

**Authors:** Helena Kandárová, Silvia Letašiová

**Affiliations:** 1 MatTek Corporation, 200 Homer Avenue, Ashland, MA 01721, USA; 2 MatTek In Vitro Life Science Laboratories, Mlynské Nivy 73, 821 05, Bratislava, Slovak Republic

**Keywords:** *in vitro*, alternative methods, ECVAM, FRAME, Validation, 3R principles

## Abstract

The development of alternative methods to animal experimentation has progressed rapidly over the last 20 years. Today, *in vitro* and *in silico* methods have an important role in the hazard identification and assessment of toxicology profile of compounds. Advanced alternative methods and their combinations are also used for safety assessment of final products. Several alternative methods, which were scientifically validated and accepted by competent regulatory bodies, can be used for regulatory toxicology purposes, thus reducing or fully replacing living animals in toxicology experimentation. The acceptance of the alternative methods as valuable tools of modern toxicology has been recognized by regulators, including OECD, FDA and EPA.

This paper provides a brief overview of the topic “alternative methods in toxicology” and focuses on pre-validated and validated alternative methods and their position in the modern toxicology.

**LIST OF ABBREVIATIONS**
CAAT: Centre for Alternatives to Animal Testing
ECVAM: European Centre For Validation of Alternative Methods
EPA: Environmental Protection Agency
ESAC: ECVAM′s Scientific Advisory Committee
FDA: Food and Drug Administration
FRAME: Fund for the Replacement of Animals in Medical Experiments
GD: Guidance Document
ICCVAM: Interagency Coordinating Committee on the Validation of Alternative Methods
JaCVAM: Japanese Center for the Validation of Alternative Methods
OECD: Organization for Economic Co-operation and Development
QSAR: Quantitative structureactivity relationship
TG: Test Guideline
ZEBET: German National Center for Evaluation and Assessment of Alternative methods

## Introduction

The development of alternative methods to animal experimentation has progressed rapidly over the last 20 years. Knowledge of alternative methods and their use in planning and conducting toxicology experiments has become essential for modern toxicologists.

**Alternative methods** (alternative toxicology tests) are methods able to:

- **reduce** the number of animals necessary in a test,
- **refine** toxicology procedures to make them less painful or stressful to laboratory animals, or,
- **replace** animals with non-animal (*in vitro*, *ex-vivo* or *in silico* systems).

These three principles, also known as the **“3Rs”,** were defined in 1959 by W.M.S. Russell and R.L. Burch in their well-crafted and scientifically valid writing on this subject, “The Principles of Humane Experimental Techniques” (Russell & Burch, 1959).

**Reduction is defined as** any decrease in the numbers of animals used to obtain information of a given amount and precision; **Refinement is defined as** any decrease in the incidence or severity of procedures applied to animals; and **Replacement is defined as** the substitution of conscious living vertebrates by non-sentient material. These three types of alternative procedures are not mutually exclusive; for example, an *in vitro* test could serve as a partial replacement for an animal test (*i.e.* it could replace the use of the animal test for certain kinds of test substances, or for a particular type and/or range of toxicological hazard). However, if the *in vitro* test were used in the context of a tiered testing strategy, it could also serve as a reduction and/or refinement alternative (*i.e.* it could reduce the number of substances tested in animals, and particularly the number of toxic substances tested) (Russell & Burch, 1959).

The 3Rs provide a strategy for a rational and stepwise approach to minimising animal use and suffering in experiments, without compromising the quality of the scientific work being undertaken. A number of useful alternative methods have been developed for evaluation of the potential toxic effects of chemicals and products since publication of the 3Rs principles. However, it still takes many years to implement these principles into the toxicology praxis. Since 1986, the concept of the 3Rs has been supported by laws in the EU that require researchers and investigators to use available alternatives before conducting *in vivo* experimentation. The **3Rs Declaration of Bologna**, which was adopted in 1999 by the Third World Congress on Alternatives and Animal Use in the Life Sciences, strongly endorsed and reaffirmed the principle of the 3Rs. Today, Reduction, Refinement and Replacement are basic tenets of EU research and other policies concerning the use of animals in scientific testing and experimentation.

The **Council Directive 86/609/EEC** on the protection of animals used for experimental and scientific purposes in article 7.2 states:

*“An experiment shall not be performed if another scientifically satisfactory method of obtaining the result sought, not entailing the use of an animal, is reasonably and practicably available*“.

Article 23 further states:

*“The Commission and Member States should encourage research into the development and validation of alternative techniques which could provide the same level of information as that obtained in experiments using animals, but which involve fewer animals or which entail less painful procedures, and shall take such other steps as they consider appropriate to encourage research in this field. The Commission and Member States shall monitor trends inexperimental methods”.*

As a response to articles 7 and 23 of the Council Directive 86/609/EEC, the **European Centre for the Validation of Alternative Methods (ECVAM)** was established in 1991. ECVAM was given the task to scientifically evaluate and validate alternative methods, to serve as an information centre, and to maintain a database on *in vitro* tests and validated methods. Once a method has undergone a formal validation, an independent peer-review process takes place. Subsequently, the ECVAM Scientific Advisory Committee (ESAC) gives advice on the scientific validity of the method. ECVAM also monitors the research projects funded by the European Commission, and maintains links with relevant platforms and associations devoted to reduction, refinement, and replacement (3Rs of animal use for scientific and regulatory purposes. Recently, two additional committees, PARERE (Network of European Regulators) and ESTAF (Institutions with vested interest in development and use of alternative methods) have been established to help ECVAM in identification of the most promising alternative method with regulatory relevance.

## Why do we need to validate alternative methods?

The validation process ensures that alternative methods developed by academic or industrial scientists will be scientifically valid and thus, eventually accepted by **regulatory authorities** for **classification and labeling**, **product approval** or **safety testing** purposes. Examples of where validated methods are required to generate toxicology data include *e.g.*:

- REACH (Registration, Evaluation, Authorization of Chemicals)
- Cosmetic directive 76/768/EEC (VII Amendment)
- Classification and Labelling of Chemicals and Transport regulations

Test **method validation** is a process based on scientifically sound principles by which the reliability and relevance of a particular test, approach, method, or process are established for a specific purpose. **Reliability** is defined as the extent of reproducibility of results from a test within and among laboratories over time, when performed using the same standardised protocol. **Relevance** of a test method describes the relationship between the test and the effect in the target species and whether the test method is meaningful and useful for a defined purpose, with the limitations identified. In brief, it is the extent to which the test method correctly measures or predicts the (biological) effect of interest, as appropriate. Regulatory need, usefulness and limitations of the test method are aspects of its relevance. New and updated test methods (both *in vivo* and *in vitro*) need to be both reliable and relevant, *i.e.*, validated (Worth & Balls, 2004; Balls *et al.*, 1990a^,^b).

**Validation criteria** for new toxicological test methods in use today were developed as collaborative efforts of lead scientists from both the *in vivo* and *in vitro* communities, regulators and other experts beginning in the early 1980's. The process was carried out under the auspices of three organizations: the Organisation for Economic Cooperation and Development (OECD), the European Centre for the Validation of Alternative Methods (ECVAM), and the Interagency Coordinating Committee on the Validation of Alternative Methods (ICCVAM). These international organizations have worked together with external experts and national organizations such as FRAME, ZEBET and CAAT on harmonizing the validation criteria so that there are no major differences between them amongst different countries and continents (Worth & Balls, 2004). Pre-validation and validation principles and criteria for how validation studies of new or updated test methods should be performed are described in detail in the OECD Guidance Document 34 (OECD, 1990).

**Figure 1:**
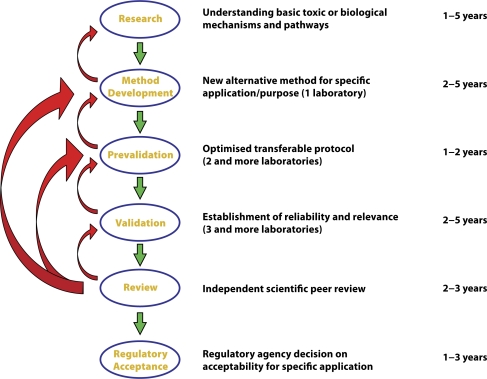
Scheme of prospective validation study.

Typically, there are two types of validation studies, prospective and retrospective validation. A prospective study involves generation of new data while a retrospective study re-assesses existing data. A typical prospective validation process is composed of 6 stages (see Figure 1). A retrospective study is usually limited to the evaluation of data submitted in a standardized and recommended form requested by particular organization performing the evaluation. A test is considered validated when its performance characteristics, advantages, and limitations have been adequately determined for a specific purpose. The measurement of a test's reliability and relevance and required for both types of validation studies.

**Predictive ability and reliability of a test is judged by:**

- Sensitivity: the percentage of positive chemicals correctly identified.
- Specificity: the percentage of negative chemicals correctly identified.
- Predictivity: the percentage of predictions for a particular classification, which were correct.
- Accuracy: the overall percentage of correct classifications.

**Other parameters assessed by the biostatistician during validation are:**

- Reproducibility within laboratories – concordance of the classifications between 3 and more independent runs in single laboratory.
- Reproducibility between laboratories – concordance of the classifications between laboratories.
- Probability for correct classification.

## Alternative methods and models used for reduction & replacement

The following systems can be used as partial or full replacements of animals in toxicology experiments: i) *in vitro* methods (primary cultures, finite lifespan cell lines, continuous cell lines, reconstructed 3D tissues), ii) *ex vivo* methods (isolated animal tissues and organs) and iii) *in silico* methods: computer simulations and mathematical models, QSAR's etc. Depending on the objective of the study, correctly selected *in vitro* methods in combination with a deep knowledge of the tested compounds (obtained from databases or computer simulations/QSARs, analytical chemistry, *etc.*) may be more appropriate for certain areas of interest than their animal counterparts.

**Advantages of *in vitro* tests:**

- controlled testing conditions,
- high level of standardisation,
- reduction of variability between experiments,
- lack of systemic effects,
- testing is fast and in most instances inexpensive,
- small amount of test material is required,
- limited amount of toxic waste is produced,
- human cells and tissues can be used,
- transgenic cells carrying human genes can be used,
- reduction of testing in animals.

**Limitations of most *in vitro* tests:**

- interactions between tissues and organs cannot be tested,
- with most *in vitro* test systems*, in vivo* dose-responses cannot be obtained for human risk assessment,
- systemic effects cannot be evaluated,
- pharmacokinetics cannot be evaluated,
- chronic effects cannot yet be tested,
- technical limitations: solubility, reaction with plastics, lack of *in vivo*-like barrier properties.

## Overview of alternative methods validated and endorsed by ECVAM, ICCVAM, OECD or other regulatory organisations

Once a method has been scientifically validated, it can enter the process of regulatory acceptance and guideline adoption. Regulatory acceptance procedures vary among countries as well as among regulatory agencies within the same country. Therefore, the Organization for Economic Cooperation and Development (OECD) promotes the harmonization of international regulatory acceptance providing the Guidance Document (GD) on the Validation and International Acceptance of New or Updated Test Methods for Hazard Assessment (OECD, 2005). Adherence to the principles defined in the OECD GD 34 increases the likelihood of the adoption of the a new or modified method.

Table 1 is adopted with minor modification from the AltTox web-site, (www.alttox.org) and provides an overview of the alternative toxicity test methods that are considered valid according to accepted international criteria. The test methods listed in this table have been judged to be scientifically valid by ECVAM, ICCVAM, JaCVAM and the OECD. Although uncommon, a test method may also be accepted for regulatory use without formal validation.

**Table 1:** Overview of validated/accepted methods (adopted with minor modifications and updates from the AltTox web-site, www.alttox.org).

End-point andMethod Name	Test Type^1^	Endorsement of Scientific Validity		Regulatory Acceptance	
		**Lead Authority**	**Subsequent Endorsement**	**National/ Regional (for methods not yet accepted internationally)**	**International acceptance**
**Acute aquatic toxicity**					
Upper threshold concentration step-down approach	*In vivo*	ESAC (2006)			
**Acute mammalian toxicity (oral)**					
Acute toxic class method	*In vivo*		ESAC (2007)		OECD TG 423 (2001)
Fixed dose procedure	*In vivo*		ESAC (2007)		OECD TG 420 (2001)
Up-and-down procedure	*In vivo*	ICCVAM (2001)	ESAC (2007)		OECD TG 425 (2006)
Normal human keratinocyte neutral red uptake (NHK NRU) assay	*In vitro*^2^	ICCVAM (2006)		US agencies (2008)	Draft OECD TG
Balb/c 3T3 neutral red uptake assay	*In vitro*^2^	ICCVAM (2006)		US agencies (2008)	Draft OECD TG
**Acute mammalian toxicity (inhalation)**					
Acute toxic class method	*In vivo*				OECD TG 436
Fixed concentration procedure	*In vivo*				Draft TG OECD 433
**Chronic toxicity**					
*Ending* 1-year dog studies of pesticides	*In vivo*	ESAC (2006)		Revised US EPA Pesticide Data Requirements	
**Dermal penetration**					
*In vitro* skin absorption methods	*In vitro ex-vivo*	OECD Expert Group (2002)			OECD TG 428 (2004)
**Endocrine mechanistic screens**					
Androgen receptor binding assay (rat prostate)	*In vitro*			OPPTS TG 890.1150 (EPA, 2009)	
Aromatase inhibition assay (human recombinant)	*In vitro*			OPPTS TG 890.1200 (EPA, 2009)	
ER-alpha transcriptional activation assay for estrogen agnoists^3^	*In vitro*				OECD TG 455 (2009)
Estrogen receptor binding assay	*In vitro*			OPPTS TG 890.1250 (EPA, 2009)	
Steroidogenesis (H295R human cell line)	*In vitro*			OPPTS TG 890.1550 (EPA, 2009)	Draft OECD TG
US EPA Tier 1 Screening Battery	*In vitro*/*In vivo*			US EPA (2009)	
**Eye corrosion**					
Bovine corneal opacity permeability (BCOP) test	*Ex-vivo*	ICCVAM (2007)	ESAC (2007) JaCVAM (2009)		OECD TG 437 (2009)
Isolated chicken eye (ICE) test	*Ex-vivo*	ICCVAM (2007)	ESAC (2007) JaCVAM (2009)		OECD TG 438 (2009)
Hen's egg test-chorioallantoic membrane (HET-CAM)	*In vitro/ Ex-vivo*			EU Competent Authorities for Dangerous Substances Directive	
Isolated rabbit eye test	*Ex-vivo*			EU Competent Authorities for Dangerous Substances Directive	
**Eye irritation**					
Cytosensor Microphysiometer modified (cytotoxicity/cell-function based *in vitro* assay)	*In vitro*	ESAC (2009)			
Cytotoxicity/cell-function based *in vitro* assay: Fluorescein Leakage	*In vitro*	ESAC (2009)			
**Genotoxicity**					
Bacterial reverse mutation (Ames) test	*In vitro*				OECD TG 471 (1997)
*In vitro* cell gene mutation test	*In vitro*				OECD TG 476 (1997)
*In vitro* chromosomal aberration test	*In vitro*				OECD TG 473 (1997)
*In vitro* micronucleus test	*In vitro*	ESAC (2006)			Draft OECD TG 487
*In vitro* sister chromatid exchange test	*In vitro*				OECD TG 479 (1986)
*In vitro* unscheduled DNA synthesis test	*In vitro*				OECD TG 482 (1986)
Saccharomyces cerevisiae gene mutation assay	*In vitro*				OECD TG 480 (1986)
Saacharomyces cerevisiae mitotic recombination assay	*In vitro*				OECD TG 481 (1986)
**Hematotoxicity: acute neutropenia**					
Colony forming unit granulocyte macrophage (CFU-GM) assay	*In vitro*	ESAC (2006)			
**Phototoxicity**					
3T3 Neutral Red Uptake Phototoxicity Test	*In vitro*	ESAC (1997)			OECD TG 432 (2004)
3T3 NRU Phototoxicity Test: Application to UV filter chemicals	*In vitro*	ESAC (1998)			OECD TG 432 (2004)
**Pyrogenicity**					
Human whole blood IL-1	*In vitro*	ESAC (2006)	ICCVAM (2008)^4^	European Pharmacopeia; US agencies	
Human whole blood IL-6	*In vitro*	ESAC (2006)	ICCVAM (2008)^4^	European Pharmacopeia; US agencies	
Human cryopreserved whole blood IL-1	*In vitro*	ESAC (2006)	ICCVAM (2008)^4^	European Pharmacopeia; US agencies	
PBMC IL-6	*In vitro*	ESAC (2006)	ICCVAM (2008)^4^	European Pharmacopeia; US agencies	
MM6 IL-6	*In vitro*	ESAC (2006)	ICCVAM (2008)^4^	European Pharmacopeia; US agencies	
Limulus amebocyte lysate (LAL) test	*In vitro*			EDQM/European Pharmacopeia;US Pharmacopeia	
**Reproductive & developmental toxicity**					
Embryonic stem cell test	*In vitro*	ESAC (2002)			
Micromass assay	*Ex vivo*	ESAC (2002)			
Whole rat embryo assay	*Ex vivo*	ESAC (2002)			
**Skin corrosion**					
Rat skin transcutaneous electrical resistance (TER)assay	*Ex vivo*	ESAC (1998)	ICCVAM (2002)		OECD TG 430 (2004)
Corrositex® noncellular membrane	*In vitro*	ICCVAM (1999)	ESAC (2000)		OECD TG 435 (2006)
EpiSkin® human skin model	*In vitro*	ESAC (1998)	ICCVAM (2002)		OECD TG 431 (2004)
EpiDerm^TM^ human skin model	*In vitro*	ESAC (1998)	ICCVAM (2002)		OECD TG 431 (2004)
EST-1000 human reconstructed epidermis	*In vitro*	ESAC (2009)			OECD TG 431 (2004)
SkinEthic^TM^ human skin model	*In vitro*	ESAC (2006)			OECD TG 431 (2004)
**Skin irritation**					
EpiSkin® skin irritation test	*In vitro*	ESAC (2007)		EU test method B.46 in COM regulation 440/2008/EC	OECD TG 439 (2010)
EpiDerm^TM^ skin irritation test	*In vitro*	ESAC (2007)^5^		EU test method B.46 in COM regulation 440/2008/EC	
EpiDerm^TM^ Modified SIT	*In vitro*	ESAC (2008)		EU test method B.46 in COM regulation 440/2008/EC	OECD TG 439 (2010)
SkinEthic RHE model	*In vitro*	ESAC (2008)		EU test method B.46 in COM regulation 440/2008/EC	OECD TG 439 (2010)
**Skin sensitization**					
Reduced LLNA	*In vivo*	ESAC (2007)	ICCVAM (2009)		
Local lymph node assay (LLNA)	*In vivo*	ICCVAM (1999)	ESAC (1999)		OECD TG 429 (2002), (2010)
Nonradiolabelled LLNA: DA	*In vivo*	ICCVAM (2009)^6^	JaCVAM (2008)		OECD TG 422A (2010)
LLNA: BrdU-ELISA	*In vivo*	ICCVAM (2009)^6^			OECD TG 422B (2010)
**Vaccine potency**					
ELISA for erysipelas vaccines batch potency testing	*In vitro*	ESAC (2002)		EDQM/European Pharmacopeia	
ELISA for human tetanus vaccines batch potency testing	*In vitro*	ESAC (2000)		EDQM/European Pharmacopeia	
Toxin binding inhibition test for human tetanus vaccines batch potency testing	*In vitro*	ESAC (2000)		EDQM/European Pharmacopeia	

1 All *in vitro* and *ex vivo* methods listed; *in vivo* methods proposed to reduce or refine animal use also listed

2 Replaces animal use for initial dose setting, but *in vivo* test required to complete assessment

3 TA assay is in process of being formally validated, but included here because of OECD TG

4 Subject to product-specific validation to demonstrate equivalence to the rabbit pyrogen test (RPT)

5 Only positive test results accepted in the 2007 endorsement

6 ICCVAM recommendations being finalized

## Conclusion

A number of validated and pre-validated methods exist that can be used as partial or full replacements of animal experiments (*e.g.* genotoxicity, testing for local toxicity effects as skin corrosion, irritation, quality control of biologicals, production of monoclonal antibodies, safety testing of final cosmetic products).

As proven by several international validation studies, alternative methods have potential to reduce the number of test animals needed for experiments or even replace the whole animal test. Testing strategies combining *in vitro*, ex vivo and *in silico* methods could be successful for areas where a single alternative method may currently be failing.

When developing alternative methods for more complex toxicity endpoints, it will be necessary to investigate the toxicology pathways and mechanisms of toxic action. At the same time, we will need to reconsider the predictive ability of the traditional animal tests and their concordance with effects observed in man. These considerations will greatly enhance our ability to produce relevant and reliable alternative methods for prediction of human health effects.
